# Supplementary material for: Endocan in Acute Leukemia: Current Knowledge and Future Perspectives
Source: Biomolecules. 2022 Mar 24;12(4):492. doi: 10.3390/biom12040492 (PMC9027427; doi:10.3390/biom12040492)
Supplement: Supplementary file 1 [file biomolecules-12-00492-s001.zip › biomolecules-1608020-supplementary.pdf]

SUPPLEMENTARY INFORMATION

Endocan in Acute Leukemia: Current Knowledge and Future Perspectives

Håkon Reikvam, Kimberley Joanne Hatfield, Øystein Wendelbo, Roald Lindås, Philippe Lassalle and Øystein Bruserud

Table S1. Serum levels of endocan in healthy controls (endocan and endocan p14 fragment levels) compared with the corresponding levels in patients with newly diagnosed acute leukemia (endocan and endocan p14 fragment levels) and acute leukemia patients with chemotherapy-induced pancytopenia (only p14 endocan fragment levels). The study included 13 patients with acute myeloid leukemia (AML) and three patients with acute lymphoblastic leukemia [3]. These results have not been and will not be presented elsewhere. All references in the table refer to the reference list in the article.

**Investigated group (endocan)**	**Number of samples[1]**	**Serum level of endocan (ng/mL; median and range)[2]**	**p-value (Mann-Whitney U test)**
Healthy controls[3]	n=20	2.08 (1.1-4.85)	
Untreated acute leukemia patients	n=13	2.82 (1.93-12.5)	p=0.0069
Untreated AML patients	n=10	3.01 (1.93-12.5)	p=0.0307

**Investigated group (p14 endocan fragment)**	**Number of samples**	**Serum level of p14 endocan fragment (ng/mL; median and range)[2]**	**p-value (Mann-Whitney U test)**
Healthy controls	n=20	1.22 (1.01-1.69)	
Untreated acute leukemia patients	n=13	0.97 (0.71-4.01)	p=0.0394
Untreated AML patients	n=10	0.98 (0.71.4.01)	Not significant
Acute leukemia patients with chemotherapy-induced cytopenia[4]			
Pancytopenia, stable	n=21	0.87 (nd-2.13)[5]	0.00034
Febrile pancytopenia (at diagnosis)	n=18	0.88 (nd-4.21)	0.00034
Pancytopenia, improvement after antibiotic treatment)	n=8	0.84 (nd-4.13)	0.0053
Acute leukemia, complete remission	n=12	0.75 (nd-1.26)	<0.00001

**Correlation analyses between endocan and p14 endocan fragment levels**	**Number of samples**		**r-value/p-value (Pearson's test)**
Patients	n=95[6]	All acute leukemia samples[6]	0.48/0.0002
Healthy controls	n=20	All healthy controls	-0.25/0.29)

1. Samples were collected after written informed consent and in accordance with the Declaration of Helsinki (Regional Ethics Committee approval REK Vest 2015/1759 and 2017/305). Serum samples were derived from 16 patients (median age 48 years, range 32-67 years; 12 males and 4 females) that represent a consecutive subset of a patient population described in detail previously [3]. Patient samples were collected at the time of diagnosis or regularly during the the period of severe pancytopenia (duration 15-25 days; before, during and following febrile neutropenia) caused by the intensive antileukemic chemotherapy [3].
2. Analysed by ELISA analyses (Biothelis; Lille, France) [3,30].
3. Twenty healthy blood donors (median age 48 years, range 24-56 years, 12 males/8 females) were examined.
4. These studies included 13 AML and three ALL patients that were investigated following 19 intensive chemotherapy cycles based on conventional cytotoxic drugs; all 19 cycles included cytarabine and/or an anthracycline) [3]. Twelve cycles represent consolidation therapy after reaching complette hematologial remission. The last seven cycles were induction cycles; morphological examination of bone marrow samples 14 days after start of chemotherapy then verified that residual leukemia could not be detected and all these patients reached complete hematological remission after the induction cycle [3]. None of the patients received targeted therapy directed against specific molecular targets, and all patients developed severe treatment-induced bone marrow failure lasting for 15-25 days [3].
5. Levels below 0.60 ng/mL could not be detected by the p14 ELISA assay (nd, not detectable).
6. Including all 60 samples from the analyses described above and 35 additional samples from the same patients collected at regularly after the diagnosis of febrile pancytopenia [3].

Table S2. A summary of systemic endocan levels in human diseases. The table describes how various human diseases alter the systemic endocan levels. All references [3,19,23,24,50] refer to the reference list in the article.

Disease	Effects on systemic endocan levels	
	**Increased**	**Decreased**
Autoimmune diseases [23,50]	Increased levels for several diseases, including psoriasis, sarcoidosis, systemic sclerosis and systemic lupus erythematosus	
Other nonmalignant diseases [23,50]	Kidney diseases, diabetes-hyperglycemia, hypertension, atherosclerosis/coronary artery disease/heart infarction, nonalcoholic fatty acid disease, hypothyreosis, obstructive sleep apnea	
Malignant diseases [3,24,50]	Increased levels have been described for several solid tumors, and high levels are often associated with aggressive disease and adverse prognosis (e.g. lung, breast, hepatocellular, renal cancer)<br>Increased levels in acute leukemia	
Intensive anticancer chemotherapy [3]		Decreased levels in patients with chemotherapy-induced pancytopenia (i.e. bone marrow failure)
Infections [3,19]	Increased in previously immunocompetent sepsis patient<br>Increased in febrile neutropenia, i.e. severe neutropenia complicated with bacterial infections.	Sepsis complicated with later development of acute respiratory distress syndrome
Covid-19 disease [83]	Moderate disease	Severe disease
Graft rejection [105,106]	Kidney graft rejection	
Allogeneic stem cell transplantation [77]		Low preconditioning/pretransplant levels associated with increased early nonrelapse mortality
